# Supplementary material for: Mass spectrometry imaging for biosolids characterization to assess ecological or health risks before reuse
Source: Nat Commun. 2023 Jul 15;14:4244. doi: 10.1038/s41467-023-40051-0 (PMC10349827; doi:10.1038/s41467-023-40051-0)
Supplement: Supplementary file 1 — Supplementary Information [file 41467_2023_40051_MOESM1_ESM.pdf]

Supplementary Information

Mass spectrometry imaging for biosolids characterization to assess ecological or health risks before reuse

Claire Villette[1], Loïc Maurer[2], Julie Zumsteg[1], Jérôme Mutterer[3], Adrien Wanko[2], Dimitri Heintz[1]*

[1] Plant Imaging & Mass Spectrometry (PIMS), Institut de biologie moléculaire des plantes, CNRS, Université de Strasbourg, 12 rue du Général Zimmer, 67084 Strasbourg, France

[2] Université de Strasbourg, CNRS, ENGEES, ICube UMR 7357, F-67000 Strasbourg, France

[3] Microscopie et imagerie cellulaire, Institut de biologie moléculaire des plantes, CNRS, Université de Strasbourg, 12 rue du Général Zimmer, 67084 Strasbourg, France

*corresponding author

Dimitri Heintz

dimitri.heintz@ibmp-cnrs.unistra.fr

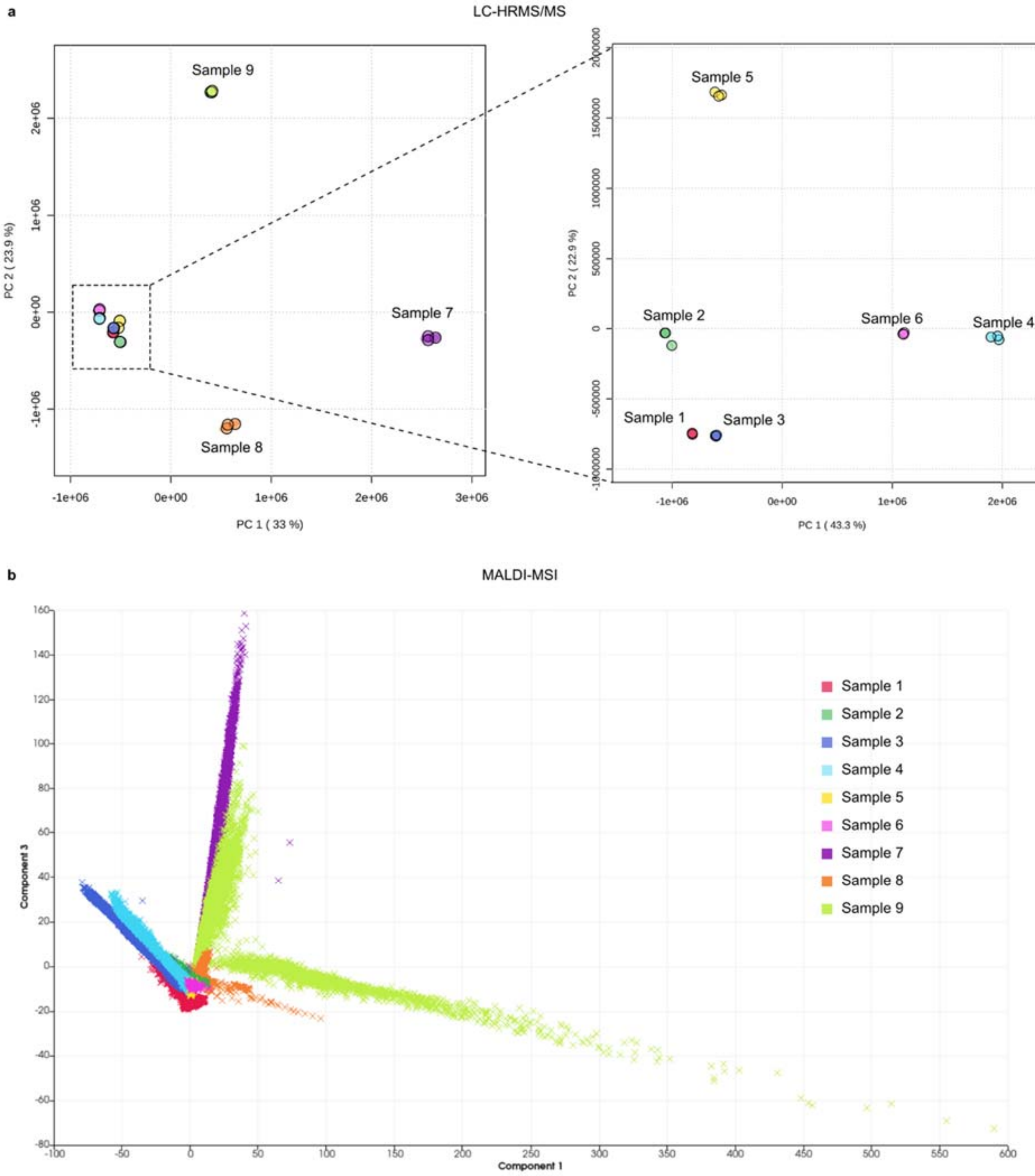

Supplementary Fig. 1. Principal component analysis (PCA) conducted on biosolid samples shows their diversity. **a**, Scores plot showing the sample diversity measured by liquid chromatography coupled to high resolution tandem mass spectrometry (LC-HRMS/MS). PCA was performed in MetaboAnalyst 5.0 on 3 replicates by biosolid (https://www.metaboanalyst.ca/). Left panel shows that samples 7, 8 and 9 are affecting most the PCA. When performed on samples 1 to 6, the PCA also shows a diversity between samples (right panel). **b**, Scores plot from the PCA conducted on biosolid samples using matrix-assisted laser desorption ionization mass spectrometry imaging (MALDI-MSI), where data points represent single mass spectra and are colored by biosolid sample (3 replicates by biosolid). PCA was performed in SCiLS Lab 2022a. PC, principal component. Source data are provided as a **Source Data** file for panel a.

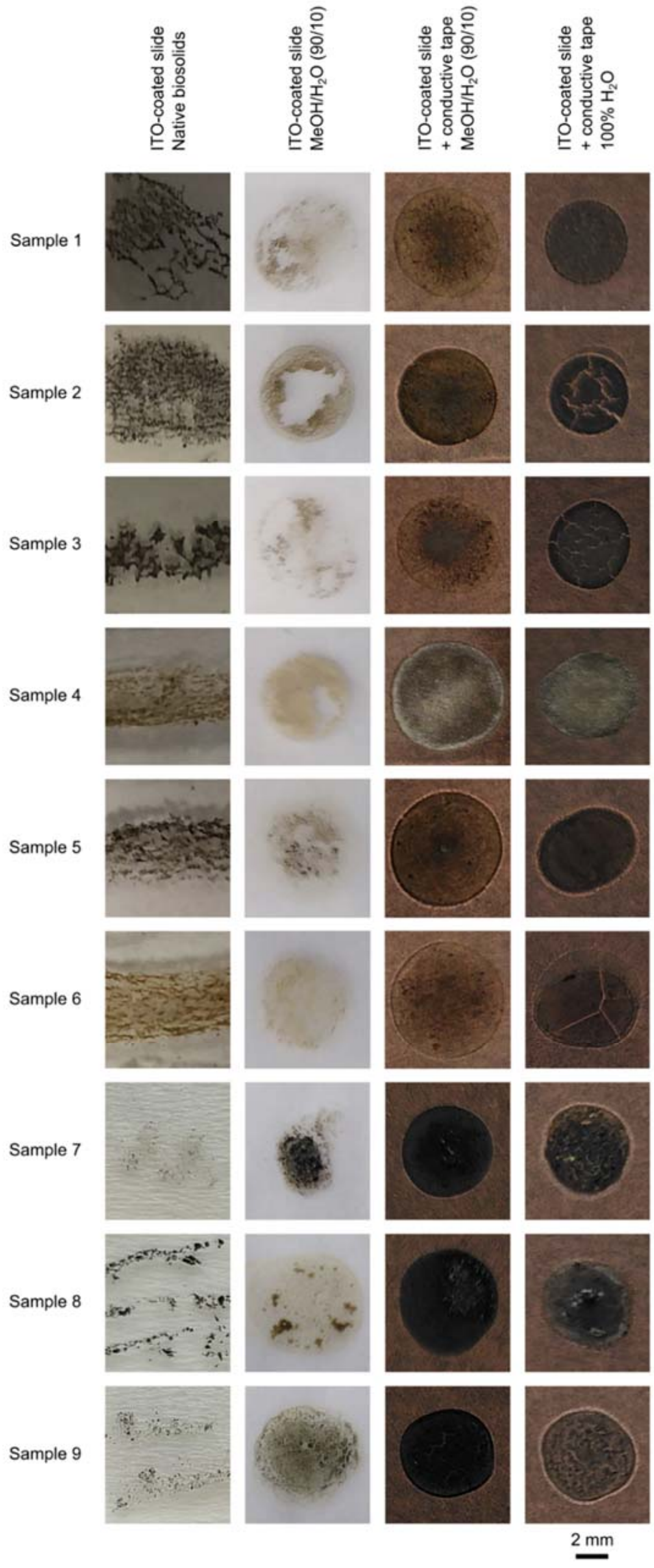

Supplementary Fig. 2. Optical images of biosolid samples optimization before matrix assisted laser desorption ionization mass spectrometry imaging (MALDI-MSI analysis). Using usual sample preparation techniques, biosolid samples tend to fall to pieces when applied on a classical MALDI slide. The use of a conductive copper tape avoids sample crumbling, whatever the solvent used for sample deposition. Three independent experiments were performed with similar results.

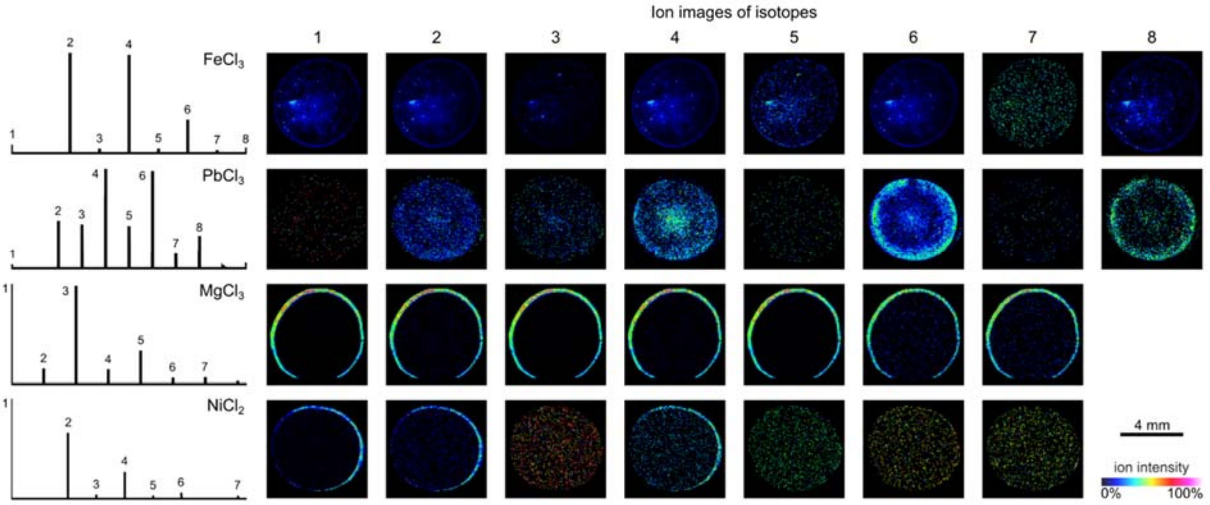

Supplementary Fig. 3. Detection of heavy metals and magnesium in biosolid samples. Heavy metals and magnesium in their chlorinated forms were successfully detected in biosolid samples. The specific isotopic patterns (left) allow for their identification, and the isotopes (numbered 1-7 or 8) could be displayed individually in the samples (right). Examples are given for $FeCl_3$ in sample 4, $PbCl_3$ in sample 1, $MgCl_3$ in sample 3, and $NiCl_2$ in sample 1. Images are displayed with a 3 ppm window. Three independent experiments were performed with similar results.

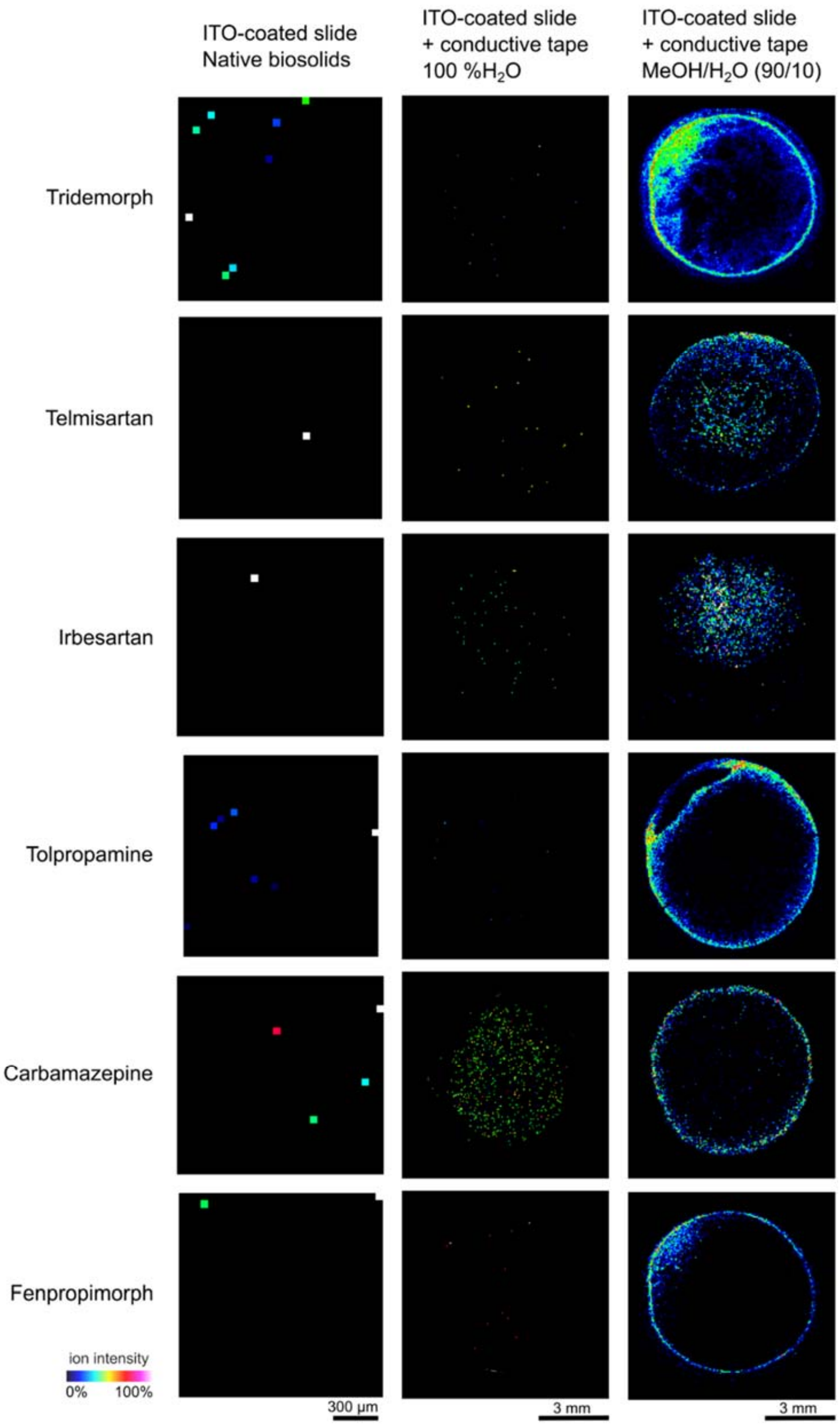

Supplementary Fig. 4. Distributions observed on native biosolids (no signal) or on biosolids deposited using solvent-forced migration with 100% H_2O or MeOH/H_2O (90/10). When using 100% H_2O, only a weak diffuse signal is observed for carbamazepine, and spare pixels are hardly visible for irbsertan and telmisartan. The use of MeOH/H_2O (90/10) helps the desorption of the compounds from biosolid matrices and creates specific accumulation areas where the compounds of interest can be detected due to the concentration effect obtained. Examples are given for fenpropimorph in sample 2, carbamazepine in sample 4, tolpropamine in sample 3, irbesartan in sample 4, telmisartan in sample 1, and tridemorph in sample 2. Images are displayed with a 3 ppm window. Three independent experiments were performed with similar results. ITO, indium tin oxide, MeOH, methanol.

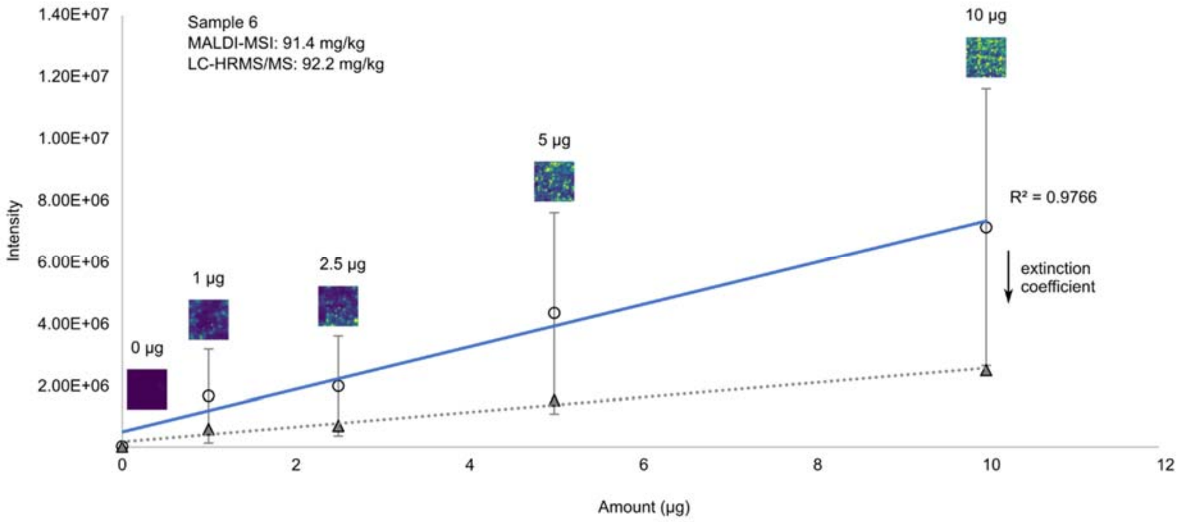

Supplementary Fig. 5. Quantification of copper in biosolids using matrix assisted laser desorption ionization mass spectrometry imaging (MALDI-MSI). A standard curve for $CuCl_2$ was established on m/z 132.86785 by spotting different amounts of copper commercial standard on an indium tin oxide (ITO)-coated slide, following previously published protocols[1]. One replicate per sample was dedicated to quantification and spotted with a known concentration of copper, allowing for the calculation of a coefficient of extinction for each sample comparable to the matrix effect in liquid chromatography coupled to mass spectrometry (LC-MS). Standard curves were derived for each sample using the calculated coefficients of extinction, allowing for the quantification of copper, the example is given for sample 6 (mg/kg dry weight). Data measured from n independent spectra acquired over the spotted surface, with n= 4,981 (0 µg); n= 2,760 (1 µg); n= 3,371 (2.5 µg); n= 7,644 (5 µg) and n= 12,566 (10 µg). Data are presented as mean values +/- standard deviation. Source data are provided as a **Source Data** file.

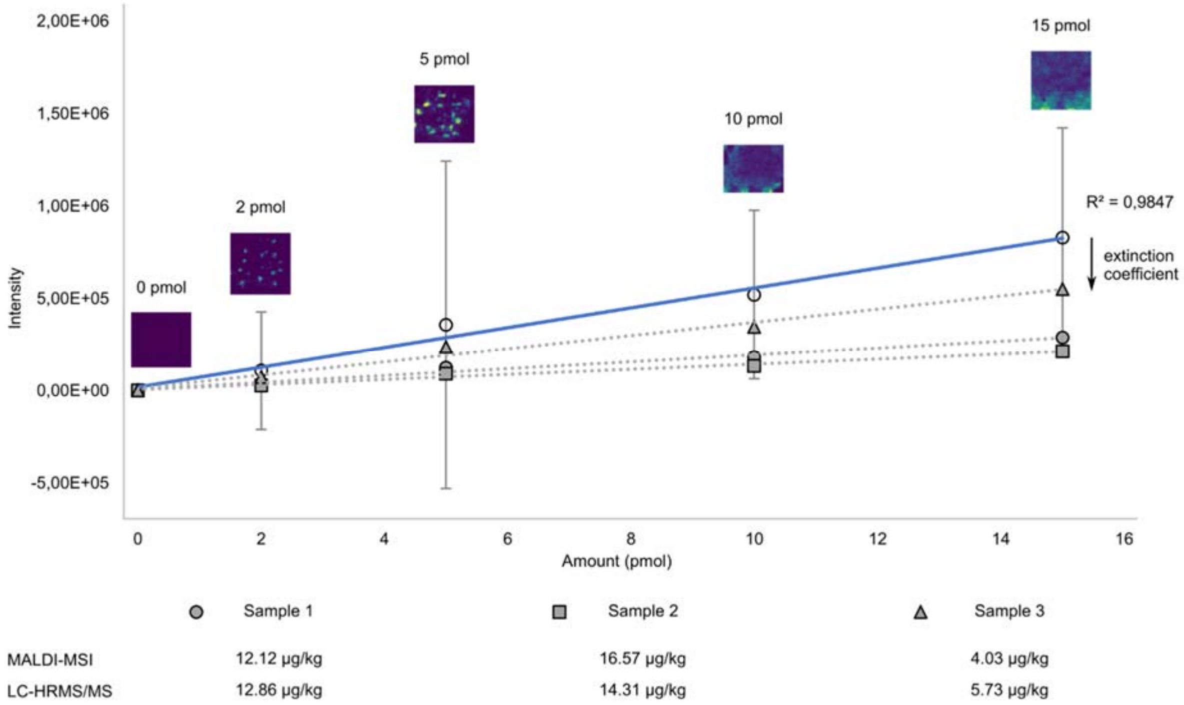

Supplementary Fig. 6. Quantification of telmisartan in biosolids using matrix assisted laser desorption ionization mass spectrometry imaging (MALDI-MSI). A standard curve was established by spotting different concentrations of telmisartan commercial standard on an indium tin oxide (ITO)-coated slide, following previously published protocols[2]. One replicate per sample was dedicated to quantification and spotted with a known concentration of telmisartan, allowing for the calculation of a coefficient of extinction for each sample comparable to the matrix effect in LC-MS. Standard curves were derived for each sample using the calculated coefficients of extinction, allowing the quantification of telmisartan in the samples (µg/kg fresh weight). Data measured from n independent spectra acquired over the spotted surface, with n= 1,362 (0 pmol); n= 1,301 (2 pmol); n= 2,207 (5 pmol); n= 1,450 (10 pmol) and n= 2,738 (15 pmol). Data are presented as mean values +/- standard deviation. Source data are provided as a **Source Data** file.

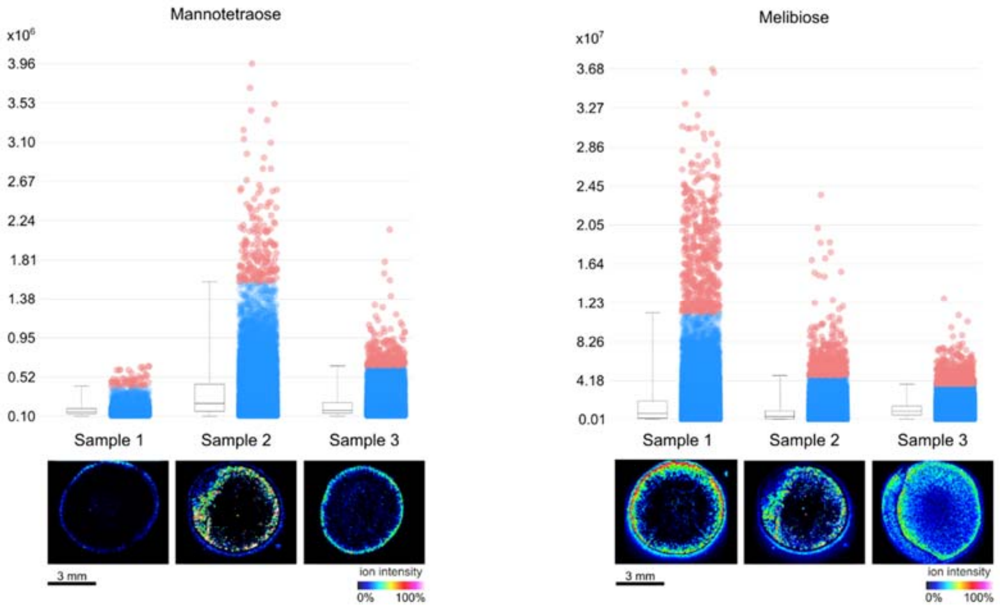

Supplementary Fig. 7. Matrix assisted laser desorption ionization mass spectrometry imaging (MALDI-MSI) can be used to describe the pathogen content of biosolids. Metabolites described as related to pathogens could be annotated and localized in the samples. According to yeast metabolome database (YMDB), mannotetraose is an oligosaccharide which "may be a unique *S. cerevisiae* (yeast) metabolite" and according to *Escherichia coli* metabolome database (ECMDB), melibiose is a sugar "produced and metabolized only by enteric and lactic acid bacteria and other microbes". The identifications were confirmed by liquid chromatography coupled to high resolution tandem mass spectrometry (LC-HRMS/MS) to level 1 of the Schymanski classification using the pure standards of the compounds. Images are displayed with a 3 ppm window, mannotetraose [M+Na]+, m/z 689.21043; melibiose [M+Na]+ m/z 365.10515. Identifications were confirmed using LC-HRMS (**Supplementary Fig. 9**). n=3 biologically independent samples. Data are presented as median values, second and third quartiles, whiskers represent lower (0%) and upper quantiles (99%).

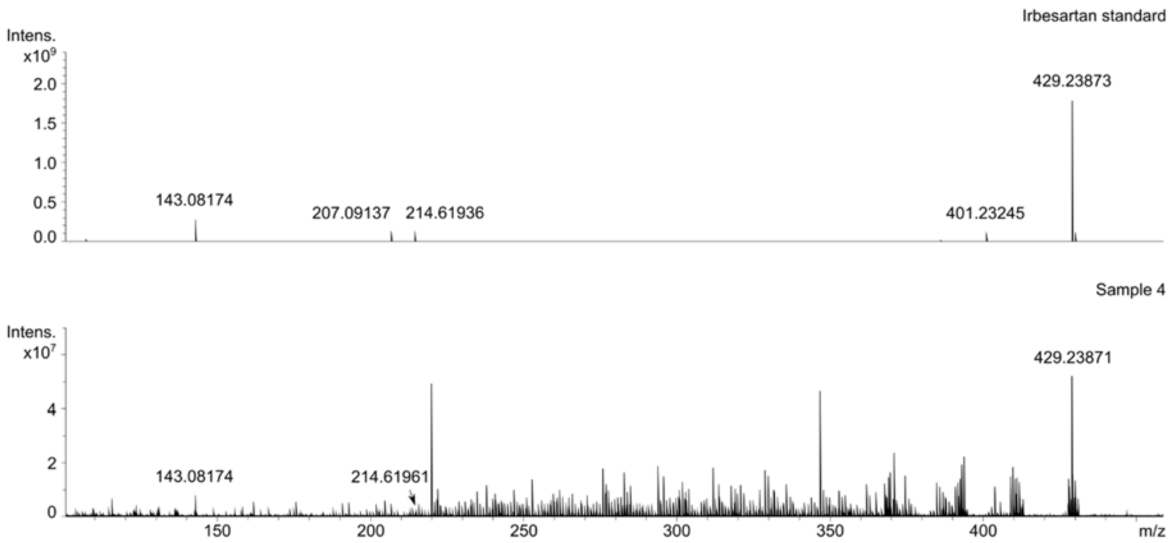

Supplementary Fig. 8. Matrix assisted laser desorption ionization mass spectrometry imaging (MALDI-MSI) fragmentation patterns of irbesartan in the commercial standard solution and in sample 4 to confirm irbesartan identification. Irbesartan was also confirmed to level 1 of the Schymanski classification using liquid chromatography coupled to tandem mass spectrometry (LC-HRMS/MS).

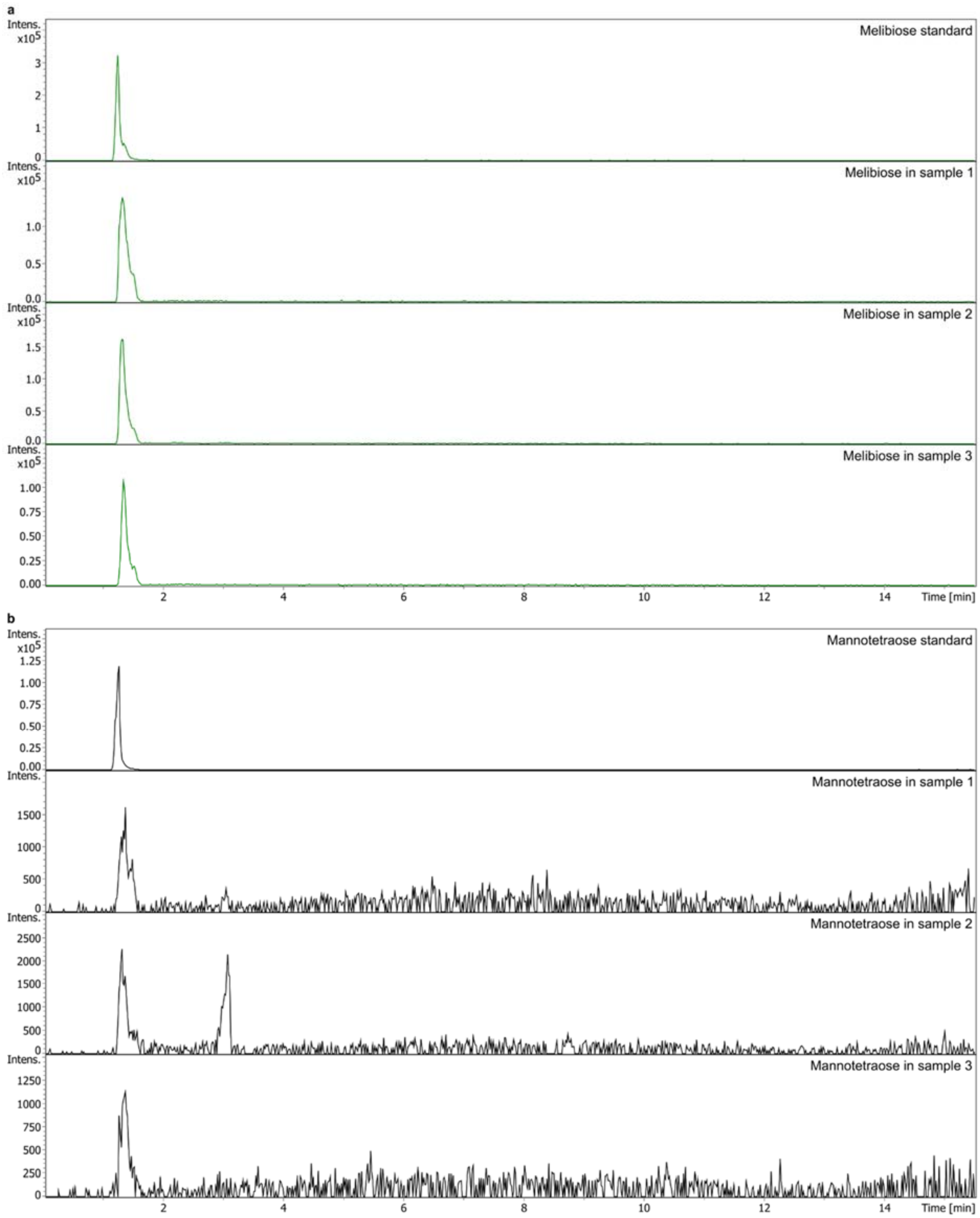

Supplementary Fig. 9. Confirmation of melibiose and mannotetraose identifications using liquid chromatography coupled to high resolution mass spectrometry (LC-HRMS). As melibiose and mannotetraose were annotated in matrix assisted laser desorption ionization mass spectrometry imaging (MALDI-MSI) based on their [M+Na]+ adduct, no fragmentation (MS/MS) was possible in MALDI-MSI. Melibiose (**a**) and mannotetraose (**b**) analytical standards at 1 µg/mL were injected in LC-HRMS, and the retention times obtained were compared to the retention times in the samples to confirm identifications. Extracted ion chromatograms (EIC) of melibiose [M+Na]+ (m/z 365.1054±0.01) and mannotetraose [M+Na]+ (m/z 689.2111±0.01) are displayed.

Supplementary Table 1. Physico-chemical parameters and metallic content measured in the samples. Metals were measured using inductively coupled plasma (ICP). Hyphens indicate that no measurement was performed for the given sample.

	1-Falkwiller	2-Lutter	3-Montiers	4-Sarrebourg	5-Hartzwiller	6-Laneuville	7-Wantzenau	8-Meistratzheim	9-Sausheim
Physico-chemical parameters (dry weight except dry matter)									
Dry matter (%)	63	63	82.9	28.9	14.7	21.8	24.4	93.4	25.5
Organic matter (%)	-	-	12	38.4	63.2	45.9	72.7	48.12	65.8
Total organic carbon (TOC, g/kg)	34.8	39.7	34.6	191.8	316.2	238	363	-	329
Kjeldahl nitrogen (TKN, g/kg)	2.8	3.5	3.9	32.6	57.8	33.8	49.7	37.9	-
TOC/TKN	12.4	11.3	8.76	5.9	5.5	7	7.3	6.4	-
Ammonium (NH_4, mg/kg)	0.15	0.5	<20	809	<1	880	7,240	3,100	4,900
Metals (mg/kg dry weight)									
Aluminium (Al)	13,300	12,600	-	-	-	-	-	-	-
Arsenic (As)	-	-	12.4	-	-	-	2.91	-	-
Cadmium (Cd)	0.75	0.95	6	1.1	0.89	0.57	0.79	1.5	1.13
Calcium (Ca)	-	-	63,600	-	-	-	47.8	-	-
Chromium (Cr)	-	-	37.8	27.6	28.6	22.8	22.9	40.4	28.7
Copper (Cu)	96.6	207	11.7	-	190	92.2	178	341.4	509
Magnesium (Mg)	-	-	7,150	-	-	-	7.97	-	-
Nickel (Ni)	37.8	35.3	20.7	17.2	25.6	16.8	13.9	31	23.9
Phosphorus (P)	0.4	0.22	1,340	-	-	-	63.1	-	-
Lead (Pb)	22.2	33.3	21.5	22.3	56	21.7	33.6	51.3	55.8
Potassium (K)	-	-	3,420	-	-	-	-	-	-
Zinc (Zn)	-	-	61.8	505	890	420	675	1,126	1259
Mercury (Hg)	<0.10	0.21	<0.10	0.51	0.72	0.31	0.79	0.8	3.6
Calcium oxide (CaO)	-	-	88,900	341,000	19,900	189,000	47,800	41,600	37,600
Magnesium oxide (MgO)	-	-	11,900	8,000	6,800	8,200	7,970	8,700	8,730
Potassium oxide (K_20)	-	-	4,110	2,900	5,500	2,480	2,820	2,800	2,090
Total phosphorus (P_2O_5)	-	-	3,080	24,900	52,100	33,500	63,100	68,200	64,200

Supplementary Table 2. Types of conductive tapes described in the literature and tested before this study.

	Teraoka Seisakusho (#796)	3M (#9711S-30)	Electron Microscopy Sciences (#77819-12)
Material	Copper	Copper	Carbon
Thickness	50 µm	30 µm	260 µm
Electrical resistance	0.03 Ω/cm²	0.05-0.1 Ω/cm²	> 1 Ω/cm²
Reference	Nakabayashi *et. al*, 2019[3]	Vandenbosch *et. al*, 2020[4]	Kelley *et. al*, 2020[5]

Supplementary Table 3. Number of annotated persistent organic pollutants (POPs) in each sample using matrix assisted laser desorption ionization mass spectrometry imaging (MALDI-MSI) on biosolids and number of annotations confirmed to level 3 to 1 of the Schymanski classification using liquid chromatography coupled to high resolution tandem mass spectrometry (LC-HRMS/MS) or gas chromatography coupled to triple quadrupole tandem mass spectrometry (GC-TQ-MS/MS). Detailed annotations are available in **Supplementary Dataset 1** and source data are provided as a **Source Data** file.

	Annotated in MALDI-MSI	MALDI-MSI + LC-HRMS/MS (Schymanski level 3)	MALDI-MSI + GC-TQ-MS/MS or LC-HRMS/MS (Schymanski level 1)
1-Falkwiller	153	17	9
2-Lutter	154	30	13
3-Montiers	182	26	10
4-Sarrebourg	153	19	8
5-Hartzwiller	101	18	8
6-Laneuville	172	28	7
7-Wantzenau	88	6	2
8-Meistratzheim	28	1	0
9-Sausheim	29	3	0
Total	499	83	28

Supplementary Table 4. Properties and environmental fate/transport for the persistent organic pollutants (POPs) annotated using matrix assisted laser desorption ionization mass spectrometry imaging (MALDI-MSI) and presented in **Fig.5** (level 1 of the Schymanski classification). Data from the CompTox Chemicals Dashboard as available on June,6th, 2022.

Name	Fenpropimorph	Carbamazepine	Tolpropamine	Irbesartan	Telmisartan	Tridemorph
Polarizability (Å^3)	37.4	27.6	32.8	49.7	61.3	37
Henry's Law (atm-m3/mole)	7.34E-07	2.24E-10	4.56E-06	2.85E-09	2.16E-08	6.52E-07
Boiling Point (°C)	345	381	333	549	653	342
Flash Point (°C)	137	210	158	345	410	129
Melting Point (°C)	102	182	66.7	240	254	30.1
Molar Refractivity (cm^3)	94.5	69.7	82.6	125	155	93.3
Molar Volume (cm^3)	327	187	259	328	415	348
Viscosity (cP)	-	20.5	12.6	-	-	6.59
Surface Tension (dyn/cm)	32.5	57.3	37.5	54.4	48.7	29
Density (g/cm^3)	0.923	1.25	0.984	1.35	1.29	0.843
Vapor Pressure (mmHg)	7.16E-06	2.04E-07	1.98E-05	1.49E-09	2.96E-11	9.30E-06
Water Solubility (mol/L)	2.2	1.75	3.3	1.25	1.55	6.32E-05
Thermal Conductivity (mW/(m*K))	131	-	136	-	-	148
Index of Refraction	1.49	1.67	1.55	1.69	1.67	1.45
LogKoa: Octanol-Air	8.85	11	8.65	10.2	11.4	8.84
LogKow: Octanol-Water	5.17	2.41	4.63	4.23	7.59	6.46
Atmos. Hydroxylation Rate (cm3/molecule*sec)	2.31E-11	1.52E-11	1.46E-11	3.33E-11	2.20E-11	1.65E-11
Biodeg. Half-Life (days)	12.6	6.54	3.65	34.8	37	8.63
Fish Biotrans. Half-Life (Km) (days)	2.36	0.66	1.67	1.56	2.04	6.18
Soil Adsorption (Koc) (L/kg)	2.36E+03	544	1.07E+03	2.15E+04	1.29E+05	1.07E+04
Bioaccumulation Factor	4.91E+02	-	-	-	-	459
Bioconcentration Factor	5.84E+02	20.8	296	22.3	42.2	427

Supplementary Table 5. Parameters used on the HTX-M5 sprayer (HTX technologies) for α-cyano-4-hydroxycinnamic acid (CHCA) and N-(1-naphthyl) ethylenediamine dihydrochloride (NEDC) matrices deposition on biosolids samples.

	CHCA	NEDC
Concentration	10 mg/mL	7 mg/mL
Solvent	70% ACN, 0.2% TFA	70% EtoH
Spray temperature	75 °C	30 °C
Passes	4	14
Flow rate	0.12 mL/min	0.06 mL/min
Velocity	1,200 mm/min	1,200 mm/min
Track spacing	3 mm	3 mm
Pattern	HH	CC
Pressure	10 psi	10 psi
Gas flow rate	3 L/min	2 L/min
Drying time	0 sec	0 sec
Nozzle height	40 mm	52.3 mm

Supplementary Table 6. Parameters used for matrix assisted laser desorption ionization mass spectrometry imaging (MALDI-MSI) data processing.

	Replicate	Number of measured pixels	Speckle size	n/Rol	Sample coverage
Sample 1	1	11,920	4x4	750	91%
	2	8,160	4x4	510	83%
	3	8,112	4x4	510	82%
Sample 2	1	11,568	4x4	730	95%
	2	13,744	4x4	860	95%
	3	12,944	4x4	810	95%
Sample 3	1	15,808	4x4	990	95%
	2	11,840	4x4	740	95%
	3	15,840	4x4	990	92%
Sample 4	1	12,288	4x4	770	92%
	2	11,056	4x4	700	95%
	3	14,720	4x4	920	95%
Sample 5	1	13,600	4x4	850	95%
	2	12,480	4x4	780	95%
	3	13,280	4x4	830	95%
Sample 6	1	15,568	4x4	980	96%
	2	15,040	4x4	940	95%
	3	14,400	4x4	900	95%
Sample 7	1	12,131	4x4	550	94%
	2	12,735	4x4	550	94%
	3	13,215	4x4	550	94%
Sample 8	1	16,646	4x4	810	94%
	2	16,171	4x4	760	94%
	3	18,308	4x4	870	95%
Sample 9	1	15,039	4x4	730	95%
	2	13,211	4x4	550	93%
	3	13,705	4x4	550	93%

Supplementary Table 7. Proposed annotations for the ions presented in **Fig. 4c**. *m*/*z*, mass to charge ratio.

Position	*m*/*z* measured	Name	Ion
A1	124.99447	2,4-dinitro Benzenesulfonic acid dihydrate	[M+H+H]2+
A2	158.99766	Bis-1,2-(chloromethoxy)ethane	[M+H]+
A3	163.18875	Ethyldimethyl(9-octadecenyl)ammonium bromide	[M+H+H]2+
A4	192.15912	Triisopropanolamine	[M+H]+
A5	203.05241	Phenyl sulfoxide	[M+H]+
A6	205.99766	2-Chloro-4-isocyanatoanisole	[M+Na]+
A7	212.95735	2-Chloro-5-nitropyrimidin-4-amine	[M+K]+
A8	220.154	Miglustat	[M+H]+
B1	227.07894	Dichloromethyl(2,4,4-trimethylpentyl)silane	[M+H]+
B2	227.11762	N-(2,6-Dimethylphenyl)-2-picolinamide	[M+H]+
B3	228.26823	1-Pentadecene	[M+NH4]+
B4	235.01495	2,4-Dichloro-6-morpholino-1,3,5-triazine	[M+H]+
B5	247.01508	4-(methylsulphonyl)-3-nitrobenzoic acid	[M+H]+
B6	248.16433	Ethylphenidate	[M+H]+
B7	249.03687	3-acetoxy-a,a,a-trifluoro-p-toluic acid	[M+H]+
B8	250.07967	2-Bromo-N,N-dibutylacetamide	[M+H]+
C1	250.13355	Epinastine	[M+H]+
C2	256.29952	Heptadecylamine	[M+H]+
C3	260.99334	1-Propanethiol, 2,3-bis[(2-mercaptoethyl)thio]-	[M+H]+
C4	261.03693	D-Fructose-1-phosphate	[M+H]+
C5	262.06279	Tolfenamic acid	[M+H]+
C6	267.97707	4-Chloro-6-nitro-o-cresyl acetate	[M+K]+
C7	268.20575	4-Heptyldiphenylamine	[M+H]+
C8	268.29952	9-Octadecen-1-amine, hydrofluoride	[M+H]+
D1	269.02178	2,4,4'-Trihydroxybenzophenone	[M+K]+
D2	269.1258	3-carboxylato-1-[2-hydroxy-3-[(4-hydroxy-2-butenyl)oxy]propyl]pyridinium sodium chloride	[M+H]+
D3	271.1801	Doxylamine	[M+H]+
D4	274.0668	N-(2,4-dinitrophenyl)-L-serine	[M+H]+
D5	276.98477	1-[3-Chloro-5-(trifluoromethyl)phenyl]-2,2,2-trifluoroethan-1-one	[M+H]+
D6	284.33092	Amines, C16-22-alkyl	[M+H]+
D7	298.11615	4-nitrophenyl N-[(1,1-dimethylethoxy)carbonyl]glycinate	[M+H]+
D8	298.2163	1-[(3,3-Diphenylpropyl)(methyl)amino]-2-methylpropan-2-ol	[M+H]+
E1	304.29968	1-(4-(trans-4-heptylcyclohexyl)phenyl)ethane	[M+NH4]+
E2	311.01919	5,5'-Oxybis(2-benzofuran-1,3-dione)	[M+H]+
E3	312.13165	4-nitrophenyl N-[(1,1-dimethylethoxy)carbonyl]-L-alaninate	[M+H]+
E4	312.36219	N-Methyldidecylamine	[M+H]+

Position	*m/z* measured	Name	Ion
E5	333.01438	Dodecafluoroheptanol	[M+H]+
E6	340.3935	N-Isodecylisotridecylamine	[M+H]+
E7	342.14214	1-(2-Hydroxy-2-phenylethyl)-3-[3-(trifluoromethyl)phenyl]urea	[M+NH4]+
E8	342.37272	1-(methyloctadecylamino)propan-2-ol	[M+H]+
F1	360.04159	3,5-dihydroxyphenyl 6-diazo-5,6-dihydro-5-oxonaphthalene-1-sulphonate	[M+H]+
F2	368.42472	1-Dodecanamine, N-dodecyl-N-methyl-	[M+H]+
F3	381.06478	3,9-diphenoxy-2,4,8,10-tetraoxa-3,9-diphosphaspiro[5.5]undecane	[M+H]+
F4	385.06308	Perfluorobutane sulfonamido amine	[M+H]+
F5	393.07569	[(4S,5S)-4-benzyl-2-oxo-5-oxazolidinyl]methyl 4-nitrobenzenesulfonate	[M+H]+
F6	401.15191	N-desmethyl diltiazem	[M+H]+
F7	423.32287	3-((5-(3-Acetoxy-2,2-dimethylpropylideneamino)-1,3,3-trimethylcyclohexyl)methylimino)-2,2-dimethylpropyl acetate	[M+H]+
F8	465.14763	Sodium 1-amino-4-[(2,6-diethyl-4-methylphenyl)amino]-9,10-dihydro-9,10-dioxoanthracene-2-sulphonate	[M+H]+

Supplementary references

1. Andersen, M. K. *et al.* Simultaneous Detection of Zinc and Its Pathway Metabolites Using MALDI MS Imaging of Prostate Tissue. *Anal. Chem.* **92**, 3171–3179 (2020).
2. Lagarrigue, M. *et al.* Localization and in situ absolute quantification of chlordecone in the mouse liver by MALDI imaging. *Anal. Chem.* **86**, 5775–5783 (2014).
3. Nakabayashi, R., Hashimoto, K., Toyooka, K. & Saito, K. Keeping the shape of plant tissue for visualizing metabolite features in segmentation and correlation analysis of imaging mass spectrometry in Asparagus officinalis. *Metabolomics* **15**, 1–5 (2019).
4. Vandenbosch, M. *et al.* Sample preparation of bone tissue for MALDI-MSI for forensic and (pre)clinical applications. *Anal. Bioanal. Chem.* **413**, 2683–2694 (2020).
5. Kelley, A. R. *et al.* Ethanol-Fixed, Paraffin-Embedded Tissue Imaging: Implications for Alzheimer's Disease Research. *J. Am. Soc. Mass Spectrom.* **31**, 2416–2420 (2020).
