# Supplementary material for: Mass spectrometry imaging for biosolids characterization to assess ecological or health risks before reuse
Source: Nat Commun. 2023 Jul 15;14:4244. doi: 10.1038/s41467-023-40051-0 (PMC10349827; doi:10.1038/s41467-023-40051-0)
Supplement: Supplementary file 3 — Description of Additional Supplementary Files [file 41467_2023_40051_MOESM3_ESM.pdf]

**Description of Additional Supplementary Files**

File Name: Supplementary Data 1
Description: Persistent organic pollutants (POPs) annotated in biosolid samples using MALDI-MSI.
